# The Characteristics of Patients Frequently Tested and Repeatedly Infected with *Neisseria gonorrhoeae*

**DOI:** 10.3390/ijerph17051495

**Published:** 2020-02-26

**Authors:** Juliën Wijers, Christian Hoebe, Nicole Dukers-Muijrers, Petra Wolffs, Geneviève van Liere

**Affiliations:** 1 Department Social Medicine and Medical Microbiology, Care and Public Health Research Institute (CAPHRI), Maastricht University Medical Center (MUMC+), P.O. Box 5800, 6202 AZ Maastricht, The Netherlands; Christian.Hoebe@ggdzl.nl (C.H.); Nicole.Dukers@ggdzl.nl (N.D.-M.); p.wolffs@mumc.nl (P.W.); genevievevanliere@gmail.com (G.v.L.); 2 Department of Sexual Health, Infectious Diseases and Environmental Health, South Limburg Public Health Service, P.O. Box 33, 6400 AA Heerlen, The Netherlands

**Keywords:** gonorrhoeae, retesting, repeat infection, sexually transmitted infections

## Abstract

We assessed whether patients repeatedly infected with *Neisseria gonorrhoeae* (NG) were different compared to patients repeatedly tested negative, to obtain insight into the characteristics of patients frequently tested and infected with NG. All patients tested for NG (n = 16,662) between January 2011 and July 2018 were included. Multivariable logistic regression analyses were performed for the outcomes “repeat NG infections” and “once NG positive and not retested” versus patients “repeatedly tested NG negative”. Of the individuals tested for NG, 0.2% (40/16,662) had repeat (≥2) NG infections, and accounted for 23% of all diagnosed NG infections. STI clinic patients, men (mostly men who have sex with men (MSM)), patients aged ≥25 years, and patients co-infected with HIV or *Chlamydia trachomatis* (CT) more often had repeat NG infections. The number of patients not retested after their initial NG diagnosis was 29.9% (92/308). Men (mostly MSM), HIV positive patients, and patients notified for sexually transmitted infections (STIs) were more often NG positive and not retested. Concluding, only 40 patients tested for NG accounted for one in four diagnosed NG infections. However, re-infections are likely to be missed among MSM and HIV positive patients, as they were mainly not retested after NG infection. It remains important to test and re-test for NG, especially in MSM, in order to halt transmission.

## 1. Introduction

*Neisseria gonorrhoeae* (NG) is one of the most commonly diagnosed bacterial sexually transmitted infection (STI) worldwide [1]. International testing guidelines advocate retesting within 3 to 12 months for all patients who test positive for NG [2,3,4]. Repeat NG infections are common; up to 40% of NG patients test positive again within one year after diagnosis [5,6,7].

Previous studies have indicated that patients with more sexual partners, men who have sex with men (MSM), and patients co-infected with other STIs are more likely to have a repeat NG infection within one year of a previous NG infection. These studies compared the characteristics of patients with one repeat infection to those patients with no repeat infection to identify high risk individuals. To date, however, it remains unknown whether the characteristics of patients with more than one repeat NG infection differ from patients repeatedly tested NG negative [8]. Differences in the characteristics between patients repeatedly infected with NG and those individuals repeatedly tested negative for NG could be indicative for different high-risk populations. However, similarities in the characteristics between those groups could suggest similar sexual networks and high risk behavior [9,10]. Furthermore, the extent to which patients with repeat NG infections account for the total number of diagnosed NG infections could provide insight into NG transmission routes [8].

In contrast, patients who are not retested after their initial NG diagnosis could also have impact on circulating STIs within a population. For example, from previous studies it is known that NG retesting rates of STI clinics and general practitioners (GPs) are relatively low, ranging from 15−22.8%, while reinfection rates are relatively high (up to 16%) [7,11]. Therefore, NG reinfections are likely to be missed leading to ongoing transmission of NG. Identifying the characteristics of patients who are once NG positive and not retested could be used to inform NG (re-)test practices and control.

Here, we compared the socio-demographic characteristics of patients with repeat NG infections with individuals repeatedly tested NG negative to identify high risk populations. Furthermore, we assessed whether the socio-demographic characteristics of patients who were once NG positive and not retested were different compared to individuals repeatedly tested NG negative to obtain insight into the population lost to care. To achieve this, we performed this study including all NG consultations in a defined geographical area in a 7.5-year timeframe.

## 2. Materials and Methods

### 2.1. Study Population

**Figure 1:**
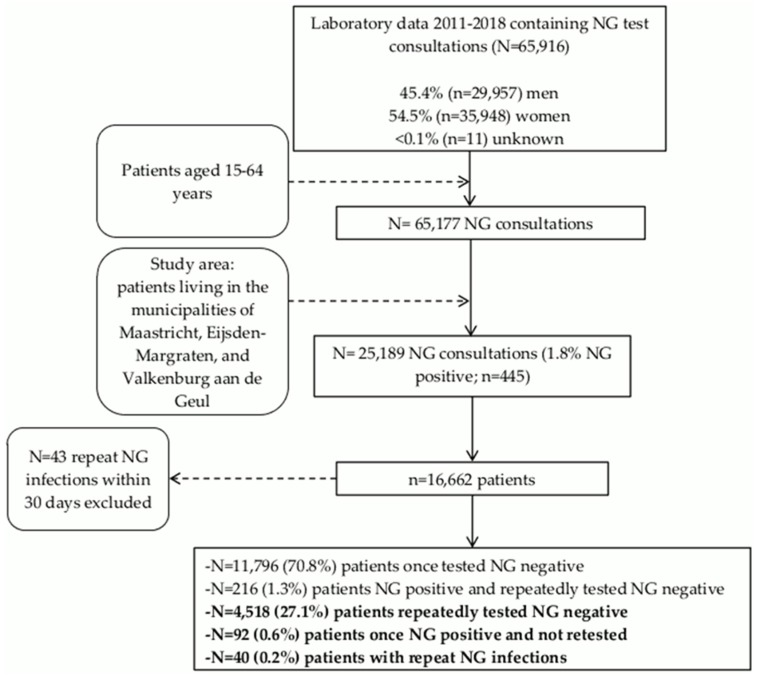
Flowchart of *Neisseria gonorrhoeae* test consultations, January 2011–July 2018.

In this cross-sectional study, all NG test consultations (n = 25,189) from January 2011 and July 2018 of 16,662 patients between 15 and 64 years old were obtained from the database of the regional Medical Microbiology Laboratory of Maastricht University Medical Center (MUMC+) (Figure 1). The database comprised consultations from all STI care providers from one geographical area in the south-eastern part of Limburg, the Netherlands. The study area included the municipalities of Maastricht, Eijsden-Margraten, and Valkenburg aan de Geul. Only data of patients living in this area were included for analyses. The distribution of NG test consultations per STI care provider were as follows: mental health care (n = 171; 0.7%), the STI clinic (n = 12,278; 48.7%), the hospital (n = 3206; 12.7%), and GPs (n = 9534; 37.8%). Of the GP practices in the study area, 81% (n = 48) send their tests to the regional laboratory ensuring acceptable laboratory coverage [7]. NG positive tests within 30 days of a previous positive NG test were excluded due to possible false−positive results (n = 43) [4].

The geographical area consisted of n = 111,162 inhabitants (hereafter residential population) between 15 and 64 years old [12]. Our main study population included all individuals tested for NG (n = 16,662).

### 2.2. Outcome Measures

Two main outcome measures were defined: (1) “*repeat NG infections*”, which were patients with ≥2 repeat NG infections within the study period, and (2) “*once NG positive and not retested*”, which were patients who were once NG positive and not retested within the study period.

### 2.3. Statistical Analyses

In our main analyses, we assessed whether the characteristics of patients classified in one of the two above described outcome measures were different compared to individuals repeatedly tested negative (≥ 2 negative NG tests) using multivariable logistic regression analyses. Determinants tested were: initial test location (mental health care facilities, STI clinic, hospital, GP), sex (men, women), age (< 25 years, ≥ 25 years), urbanization (rural, urban), HIV co-infection (not tested, yes, no), and any CT co-infection (yes, no) during the study period. We noticed that 5.9% (n = 977) of the patients tested for NG changed STI care provider after their initial test (Supplementary File 1). Therefore, the determinant ‘initial test location’ was based on the STI care provider where the patient was firstly tested for NG. The determinant urbanization was defined according to Statistics Netherlands: areas with ≥1500 addresses per km^2^ were categorized as “urban” and areas with <1500 addresses per km^2^ were categorized as “rural” [12].

Our secondary study population included STI clinic visitors tested for NG (n = 8022). Analyses were performed for determinants only available for the STI clinic population and included: the maximum number of sex partners in the past 6 months prior to a consultation of a patient in the entire study period (unknown, 0−1, 2−3, ≥ 4), any urogenital symptoms during the study period (unknown, yes, no), any proctitis during the study period (unknown, yes, no), any oropharyngeal symptoms during the study period (unknown, yes, no), any notification for STI during the study period (unknown, yes, no), and transmission group (men who have sex with women (MSW), MSM and women).

We calculated which proportion of the residential population (n = 111,162) were tested for NG, once infected, and repeatedly infected with NG.

Determinants with *p* < 0.10 in the univariable logistic regression models were included in the multivariable model. Odds ratios and 95% confidence intervals (CI) were calculated and presented. All analyses were performed using SPSS V24 (IBM SPSS Statistics for Windows, IBM Corporation, Armonk, New York, NY, USA). A *p*-value of < 0.05 was considered statistically significant.

Additionally, we visualized residence areas of patients (based on their 4-digit postal code), who were classified in one of the two earlier mentioned outcome measures, in a geographical map to inform potential outreach activities, and targeted testing using Qgis 2.18.28 [13].

### 2.4. Ethics Statement

The medical ethics committee of the Maastricht University Medical Center (Maastricht, the Netherlands) approved this study (METC 2017−0251) and waived the need for consent to be collected from participants. Since retrospective data originated from regular care and were analyzed anonymously, no further informed consent for data analysis was obtained.

## 3. Results

### 3.1. NG Testing and Positivity in the Residential Population

Of the 111,162 people residing in the study area, 15.0% (n = 16,662) were tested for NG. 0.3% (n = 348) tested positive at least once; 0.3% (n = 308) were diagnosed with one NG infection; and 0.04% (n = 40) with two or more NG infections.

Within the individuals tested for NG (n = 16,662), the vast majority (70.8%) were tested once and found NG negative (n = 11,796), and 0.2% (n = 40) were repeatedly (≥ 2) infected with NG (Figure 1). The characteristics of all individuals tested for NG are presented in Table 1.

Of the 308 patients once infected with NG, 29.9% (n = 92) were not retested after their initial NG diagnosis. All 348 NG positive patients contributed to 402 NG infections. Of these 402 NG infections, repeat NG infections (≥ 2 NG infections) accounted for 23.4% (n = 94).

### 3.2. Characteristics of Patients with Repeat NG Infections

The characteristics of patients repeatedly infected with NG (≥ 2 NG infections) (n = 40) were compared to the characteristics of patients repeatedly tested negative for NG (n = 4518). In multivariable analyses, patients repeatedly infected with NG were more likely STI clinic patients, men, aged ≥ 25 years, not tested for HIV, co-infected with HIV, or co-infected with CT.

In our secondary analyses among STI clinic visitors, patients repeatedly infected with NG (n = 30) were more likely having urogenital symptoms, having proctitis, notified for STIs or MSM (Table 2).

### 3.3. Characteristics of Patients Once Tested NG Positive and Not Retested

The characteristics of patients who tested once NG positive and who were not retested (n = 92) were compared to the characteristics of patients who repeatedly tested negative for NG (n = 4518). In multivariable analyses, these patients less often visited the STI clinic or hospital, thus more often visited the GP. They were more often men, not tested for HIV or HIV positive (Table 1).

In our secondary analyses among STI clinic visitors, patients once NG positive and not retested (n = 37) were more often notified for STIs, MSW or MSM (Table 2).

### 3.4. Geographical Mapping

The four-digit postal code of the patient was used to explore the location of patients infected with NG for potential outreach activities.

**Figure 2:**
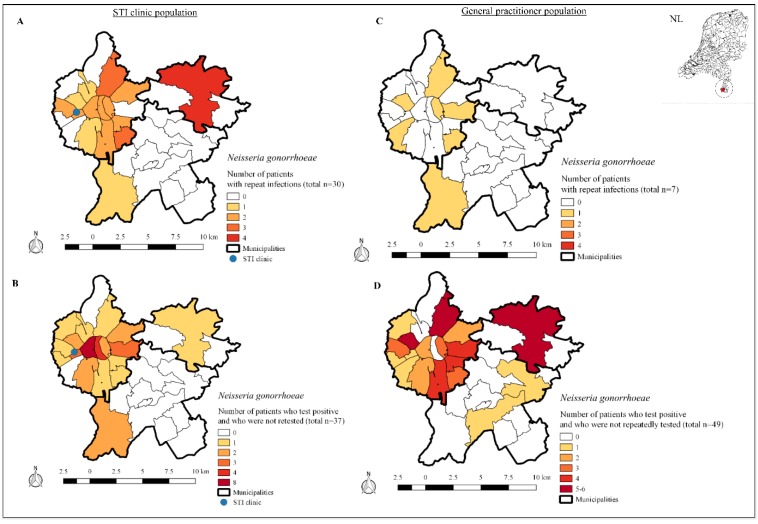
Geographical maps of patients infected with *Neisseria gonorrhoeae*.

For the STI clinic population, three local areas were visualized where ≥3 patients with repeat NG infections reside (Figure 2A). Two local areas nearby the STI clinic were visualized where ≥4 patients, who tested positive and who were not retested, reside (Figure 2B).

For the GP population, seven areas were visualized where one patient with repeat NG infections reside (Figure 2C). Six local areas were visualized where ≥ 4 patients, who were NG positive and who were not retested, reside (Figure 2D).

Geographical maps including four-digit postal code areas where patients with repeat *Neisseria gonorrhoeae* infections reside (A and C), and four-digit postal code areas where patients who test positive for *Neisseria gonorrhoeae* and who were not retested reside (B and D) stratified for the STI clinic population and general practitioner population. Location of the study area, as part of the Netherlands, is provided in the right upper corner.

## 4. Discussion

This study provides an overview of individuals tested for NG at different STI care providers in a defined geographical area. We showed that 40 patients (0.2% of the patients tested for NG) were repeatedly infected with NG and accounted for one in four diagnosed NG infections. The vast majority of these patients were diagnosed at the STI clinic and were MSM. These patients are likely at highest risk for acquiring and transmitting NG. Nevertheless, focus should also be on men (including mostly MSM, but also MSW), patients co-infected with HIV, and patients notified for STIs, as they were more often once NG positive without repeat testing, potentially leading to missed repeat NG infections and ongoing transmission of NG.

Strengths of the study are the inclusion of all NG consultations of STI care providers in a defined geographical area to obtain insight in transmission of NG. Due to inclusion of all tests by the STI clinic and hospital specialists and the high coverage of GP data (81%), underestimations of NG tests seem unlikely [7]. A further strength is the timeframe of seven and a half years to partly prevent underestimations of repeat infections [8]. The additional analyses among STI clinic visitors allowed the assessment of additional sexual behavior determinants to draw conclusions specified for STI clinic visitors. Furthermore, we visualized geographical areas where patients with repeat NG infections and patients who were not retested after NG diagnosis reside. Such geographical visualizations could inform local outreach activities and targeted testing.

A limitation of the study was that information on reasons for testing was unavailable. These may include financial reasons. For example, STI tests at the GP are within patients’ deductibles in healthcare insurance, whereas STI tests at the STI clinic are free of charge for risk groups like young people (aged *<* 25 years), MSM, and commercial sex workers. Such reasons for testing could provide more insight into why patients are (not) repeatedly tested. Furthermore, the additional determinants (sexual risk behavior and symptoms) assessed for the STI clinic population were not available for the mental healthcare, GP, and hospital population. Therefore, we could not assess whether men visiting these care providers were MSM or not. However, based on additional analyses among STI clinic patients, it is likely that men diagnosed with NG by GPs are mostly MSM.

Another study observed that 28% of all diagnosed STIs were repeat (≥ 2) STIs [8]. The authors concluded that a relatively small group of patients repeatedly infected with STIs likely have disproportionally high impact on circulating STIs within a population, the so called “core group” [8]. They included more STIs in their case definition including syphilis, NG, and CT. However, we believe core group transmission is STI specific. For example, 76% of the NG cases in the Netherlands are diagnosed in MSM which makes core group transmission more likely as compared to CT, which is a population disease affecting the general population such as men, women, and people aged < 25 years [14]. Furthermore, the majority of patients with repeat NG infections were living in the municipality of Maastricht, which is in general a more urbanized area compared to the other municipalities, suggesting that high risk NG groups mainly cluster in cities. As MSM are disproportionally affected by NG in the Netherlands and Australia, and only 40 patients accounted for one in four diagnosed NG infections, core group transmission is likely [14,15].

Despite patients with repeat testing and repeat infections being at high risk for transmitting and acquiring NG, we point attention to the fact that one in three NG patients were once NG positive and not retested and, therefore, lost to care. This enables ongoing transmission as repeat NG infections are common among patients retesting within one year [7,13]. The Centers for Disease Control and Prevention (CDC) recommends that all men and women infected with NG should be retested three months after treatment [4]. Retesting NG positive patients is an effective control strategy and can be used to enhance population-based prevention [4]. Despite that retesting is advised in many international guidelines [2,3,4], retesting rates remain typically low [7,13], indicating a need for improvement among STI care providers. Moreover, areas with relatively many patients, who were not retested after NG diagnosis, were located nearby the STI clinic. This geographical information provides a window of opportunity to inform targeted testing and outreach activities.

STI clinic patients with urogenital symptoms or proctitis had more often repeat NG infections. Studies have shown a higher NG bacterial load among symptomatic men suggesting higher transmission potential and likely clinical relevance [16,17]. Therefore, men with symptoms suggestive of NG should be encouraged to present early for treatment to prevent ongoing transmission [16].

The highest NG positivity rates are found among STI clinic patients notified for NG (29.8% in women, 19.0% in heterosexual men, and 30.9% in MSM) [14]. Notably, STI clinic patients notified for STIs were repeatedly infected with NG, but also lost to care indicating the essential role of partner management for targeting, testing, and treating this high-risk population.

## 5. Conclusions

Only 40 patients with repeat NG infections (0.2% of the individuals tested for NG) accounted for one in four NG infections. These patients were mainly STI clinic patients, MSM, and patients co-infected with CT or HIV. STI clinic patients with urogenital symptoms, proctitis, or notified for STIs had more often repeat NG infections, arguing for higher transmission potential. Therefore, it remains important to test and treat STI clinic patients, and in particular MSM for repeat NG infections. However, focus should also be on GP patients, men (including MSM and MSW), patients co-infected with HIV, and patients notified for STIs, as they were more often not retested after NG diagnosis, indicating missed repeat NG infections and ongoing transmission of NG.

## Figures and Tables

**Table 1:** Determinants associated with “repeat NG infections”, “Once NG positive and not retested”, and “once NG positive and repeatedly tested NG negative” between January 2011 and July 2018.

	All Individuals Tested for NG	Frequently Tested NG Negative	Repeat NG Infections			Once NG Positive and not Retested		
		% (n)	% (n)	OR (95%CI)	Adj. OR (95%CI)	% (n)	OR (95%CI)	Adj. OR (95%CI)
Overall % (n)	100 (16,662)	100 (4,518)	100 (40)			100 (92)		
**Initial test location**								
Mental healthcare	0.7 (121)	0.6 (28)	2.5 (1)	8.55 (1.02−71.79)	8.47 (0.91−78.59)	1.1 (1)	1.22 (0.16−9.16)	0.53 (0.07−4.20)
STI clinic	48.1 (8022)	53.6 (2,422)	75.0 (30)	2.96 (1.30−6.76)	3.29 (1.36−8.00)	40.2 (37)	0.52 (0.34−0.80)	0.51 (0.33−0.79)
Hospital	13.3 (2216)	8.7 (393)	5.0 (2)	1.22 (0.25−5.88)	0.33 (0.06−1.88)	5.4 (5)	0.44 (0.17−1.10)	0.31 (0.11−0.85)
General practitioner	37.8 (6303)	37.1 (1675)	17.5 (7)	1	1	53.3 (49)	1	1
**Sex**								
Men	37.1 (6182)	33.8 (1529)	87.5 (35)	13.68 (5.35−35.00)	8.38 (3.11−22.55)	72.8 (67)	5.24 (3.30−8.33)	7.09 (4.33−11.62)
Women	62.9 (10,480)	66.2 (2989)	12.5 (5)	1	1	27.2 (92)	1	1
**Age**								
<25 years	46.5 (7748)	44.9 (2028)	22.5 (9)	1	1	45.7 (42)	1	
≥25 years	53.5 (8914)	55.1 (2490)	77.5 (31)	2.81 (1.33−5.91)	3.10 (1.33−7.23)	54.3 (50)	0.97 (0.64−1.47)	
**Urbanization**								
Rural	31.1 (5182)	29.6 (1337)	47.5 (19)	2.15 (1.15−4.01)	1.93 (0.97−3.82)	37.0 (34)	1.39 (0.91−2.14)	
Urban	68.9 (11474)	70.4 (4516)	52.5 (21)	1	1	63.0 (58)	1	
**HIV co-infection**								
Not tested	42.1 (7014)	25.4 (1149)	20.0 (8)	1.15 (0.50−2.62)	2.75 (1.13−6.70)	41.3 (38)	2.27 (1.48−3.50)	3.99 (2.52−6.32)
Yes	1.5 (243)	1.5 (70)	50.0 (20)	28.28 (13.31−60.09)	23.89 (9.19−62.11)	6.5 (6)	5.89 (2.44−14.22)	5.38 (2.04−14.16)
No	56.4 (9405)	73.0 (3299)	30.0 (12)	1	1	52.2 (48)	1	1
**Any CT co-infection** ^a^								
Yes	12.5 (2076)	25.5 (1151)	65.0 (26)	5.43 (2.83−10.44)	4.91 (2.43−9.96)	29.3 (27)	1.22 (0.77−1.91)	
No	87.4 (14,558)	74.5 (3367)	35.0 (14)	1	1	70.7 (65)	1	

^a^ The number of patients not tested for CT was 28. All these patients were single tested for NG and NG negative. Abbreviations: CT, *Chlamydia trachomatis*, NG, *Neisseria gonorrhoeae*; HIV, Human immunodeficiency virus; STI, sexually transmitted infection; OR, odds ratio; CI, confidence interval; Adj., adjusted; na, not applicable.

**Table 2:** Secondary analyses among only the STI clinic population tested for NG between January 2011 and July 2018 including determinants associated with “repeat NG infections”, and “once NG positive and not repeatedly tested” using patients frequently tested NG negative as the reference group.

	All Individuals Tested for NG	Frequently Tested NG Negative	Repeat NG Infections			Once NG Positive and not repeatedly tested		
	% (n)	% (n)	% (n)	OR (95%CI)	Adj. OR (95%CI)	% (n)	OR (95%CI)	Adj. OR (95%CI)
Overall % (n)	100 (8022)	100 (2422)	100 (30)			100 (37)		
**Maximum number of sex partners**								
Unknown	2.8 (228)	1.4 (34)	3.3 (1)	8.50 (0.52−139.01)	na	2.7 (1)	1.21 (0.15−10.17)	
0−1	25.8 (2068)	11.9 (289)	3.3 (1)	1	1	18.9 (7)	1	
2−3	42.6 (3420)	44.1 (1068)	20.0 (6)	1.62 (0.20−13.54)	0.64 (0.07−5.82)	29.7 (11)	0.43 (0.16−1.11)	
≥4	28.7 (2306)	42.6 (1031)	73.3 (22)	6.17 (0.83−45.95)	1.08 (0.13−9.04)	48.6 (18)	0.72 (0.30−1.74)	
**Any urogenital symptoms**								
Unknown	4.6 (371)	2.3 (55)	3.3 (1)	4.45 (0.46−43.48)	3.23 (0.04−265.73)	10.8 (4)	4.85 (1.50−15.74)	19.22 (4.29−86.05)
Yes	54.0 (4329)	67.4 (1633)	86.7 (26)	3.90 (1.18−12.91)	8.31 (2.40−28.85)	59.5 (22)	0.90 (0.43−1.86)	2.19 (0.99−4.80)
No	41.4 (3322)	30.3 (734)	10.0 (3)	1	1	29.7 (11)	1	1
**Any Proctitis**								
Unknown	4.6 (371)	2.3 (55)	3.3 (1)	2.70 (0.35−20.93)	3.23 (0.04−265.73)	10.8 (4)	5.41 (1.83−15.95)	19.22 (4.29−86.05)
Yes	8.5 (680)	11.8 (285)	50.0 (15)	7.83 (3.74−16.39)	3.01 (1.32−6.85)	13.5 (5)	1.31 (0.50−3.41)	0.93 (0.34−2.55)
No	86.9 (6971)	86.0 (2082)	46.7 (14)	1	1	75.7 (28)	1	1
**Any oropharyngeal symptoms**								
Unknown	4.6 (371)	2.3 (55)	3.3 (1)	1.80 (0.24−13.66)		10.8 (4)	4.65 (1.59−13.62)	19.22 (4.29−86.05)
Yes	10.4 (831)	15.9 (386)	30.0 (9)	2.31 (1.04−5.11)		5.4 (2)	0.33 (0.08−1.39)	0.24 (0.06−1.04)
No	85.0 (6820)	81.8 (1981)	66.7 (20)	1		83.8 (31)	1	1
**Any notification for STI**								
Unknown	3.5 (278)	2.2 (54)	3.3 (1)	2.71 (0.35−21.05)	na	2.7 (1)	1.68 (0.22−12.68)	0.45 (0.04−4.92)
Yes	14.0 (1127)	19.4 (469)	53.3 (16)	4.98 (2.38−10.43)	3.19 (1.43−7.14)	40.5 (15)	2.89 (1.48−5.65)	2.21 (1.10−4.44)
No	82.5 (6617)	78.4 (1899)	43.3 (13)	1	1	56.8 (21)	1	1
**Transmission group**								
MSW	30.1 (2416)	21.6 (523)	6.7 (2)	1.95 (0.32−11.68)	2.05 (0.33−12.76)	29.7 (11)	4.59 (1.77−11.90)	5.14 (1.94−13.62)
MSM	11.2 (902)	15.4 (372)	83.3 (25)	34.21 (10.27−113.90)	41.16 (10.58−160.23)	51.4 (19)	11.14 (4.65−26.70)	15.22 (5.91−39.18)
Women	58.6 (4704)	63.0 (1527)	10.0 (3)	1	1	18.9 (7)	1	1

Abbreviations: NG, *Neisseria gonorrhoeae*; HIV, Human immunodeficiency virus; STI, sexually transmitted infection; OR, odds ratio; CI, confidence interval; Adj., adjusted.

## Supplementary Materials

The following are available online at https://www.mdpi.com/1660-4601/17/5/1495/s1.
