# Supplementary material for: The Characteristics of Patients Frequently Tested and Repeatedly Infected with Neisseria gonorrhoeae
Source: Int J Environ Res Public Health. 2020 Feb 26;17(5):1495. doi: 10.3390/ijerph17051495 (PMC7084364; doi:10.3390/ijerph17051495)
Supplement: Supplementary file 1 [file ijerph-17-01495-s001.pdf]

Supplementary file 1. Patients who changed from STI care provider after initial test, 2011-2018

**Changed from STI care provider after initial test**	***Neisseria gonorrhoeae*** **% (n)**
Not changed from STI care provider	94.1 (15,685)
From STI clinic to GP	1.8 (307)
From GP to STI clinic	1.0 (160)
From GP to hospital	1.4 (241)
From hospital to GP	0.8 (135)
From STI clinic to hospital	0.5 (87)
From hospital to STI clinic	0.2 (33)
From mental health care to GP	<0.1 (5)
From GP to mental health care	<0.1 (3)
From mental health care to STI clinic	<0.1 (2)
From mental health care to hospital	<0.1 (3)
From STI clinic to mental health care	<0.1 (1)
Total:	100 (16,662)
